# The Relationship between Cardiovascular Autonomic Dysfunction and Ocular Abnormality in Chinese T2DM

**DOI:** 10.1155/2017/7125760

**Published:** 2017-06-01

**Authors:** Dandan Wang, Baiyu Shen, Chunrong Wu, Yanyan Xue, Yanjun Liu

**Affiliations:** ^1^Department of Endocrinology, The 306th Teaching Hospital of People's Liberation Army, Peking University, Beijing, China; ^2^Department of Endocrinology, The 306th Hospital of People's Liberation Army, Beijing, China

## Abstract

**Objective:**

This study aims to explore the relationship between autonomic nerve dysfunction—assessed by cardiovascular autonomic neuropathy risk score (CAN-RS)—and ocular abnormality in Chinese type 2 diabetes mellitus (T2DM).

**Method:**

This is a cross-sectional study. A total of 335 subjects with T2DM were enrolled. The state of visual acuity, the lens, the vitreous, and the fundus were tested by professional ophthalmic instruments. The electrochemical skin conductance (ESC) of the hands and feet was measured by SUDOSCAN, from which a cardiovascular autonomic neuropathy risk score (CAN-RS) was calculated. Receiver operating characteristic (ROC) curves were drawn to evaluate the feasibility and accuracy of CAN-RS in diabetic oculopathy screening.

**Results:**

Abnormalities of the lens, vitreous, and fundus accounted for 7.8%, 5.1%, and 9.9%, respectively, in this study. The means of hands and feet ESC were higher than 60 *μ*S, and CAN-RS was 33.1 ± 14.8%. In logistic regression analysis, CAN-RS was positively associated with lens (OR = 1.055, *P* < 0.001) and vitreous (OR = 1.044, *P* < 0.01) abnormality. The area under ROC to detect lens and vitreous abnormality was 0.713 and 0.725, respectively.

**Conclusion:**

CAN-RS, a cardiac autonomic nerve dysfunction index calculated by SUDOSCAN, may be a promising index for lens and vitreous abnormality screening in T2DM patients. Further studies are needed to confirm the conclusion.

## 1. Introduction

Diabetic oculopathy is one of common complications in diabetics. Nearly all of the oculopathies can occur in diabetics, including retinopathy, uveitis, cataract, vitreous opacity, glaucoma, and optic neuropathy [1]. The Chinese Diabetes Committee reported that the blindness rate in diabetics was 25-fold higher than that in nondiabetics. So it is important to detect and diagnose diabetic oculopathy early.

Autonomic neuropathy and microangiopathy usually develop in parallel in diabetic patients. Many studies have demonstrated that retinopathy is related to cardiac autonomic neuropathy (CAN) [2–4]. Diabetic retinopathy (DR) may be a strong predictor for CAN [5, 6]. Evidence has shown that the close association between CAN and retinopathy likely stems from changes in the vasomotor control of the small vessels [7]. In these studies, the diagnostic method for CAN mainly refers to cardiovascular autonomic reflex tests (CARTs) [8], which are cumbersome, time consuming, and require strict cooperation. As for DR, the principal tools are funduscope and fluorescein angiography, which require considerable professional skill and time. Under these conditions, it is difficult to screen non- or poorly compliant patients in daily clinical practice, especially in a resource-poor medical environment. It is supposed that there may be other relatively simple substitutes for CARTs such as heart rate variability, postural blood pressure changes, baroreflex sensitivity, and cardiac radionuclide imaging [9] as well as the exercise-related heart rate changes [10, 11], and the relationship between these alternative methods and diabetic oculopathy should be verified further.

Damage to small nerve fibers may develop in the early course of diabetes and can be assessed by sudomotor function testing [10]. SUDOSCAN (Impeto Medical, France) is a recently developed sudomotor function test of the electrochemical skin conductance (ESC) of the hands and feet, which has been used widely in early diagnosis of symmetrical diabetic neuropathy [12, 13]. SUDOSCAN can also be used for the efficient screening of CAN by means of its proprietary cardiovascular autonomic neuropathy risk score (CAN-RS) [13–15], which is derived from ESC, HbA1c, age, and BMI. Many studies have been published which investigate the relationship between CAN-RS and diseases such as metabolic syndrome [16] and arterial stiffness [17], but its association with diabetic oculopathy remains unexplored. The previous studies usually focus mainly on DR in diabetic patients; however, other types of ocular abnormality are rarely investigated.

This study aims to examine the relationship between CAN-RS and diabetic oculopathy (including fundus lesion and other eye abnormalities) and to further explore whether CAN-RS can be used to screen for diabetic oculopathy.

## 2. Materials and Methods

### 2.1. Subject

Type 2 diabetes patients above 18 years of age were enrolled from the outpatient population in the People's Liberation Army (PLA) Diabetes Diagnosis & Treatment Center. Exclusion criteria included patients with tumors, thyroid disease, immunological diseases, and autonomic nervous function disorders with pathogenies other than diabetes. Subjects with autonomic nerve function asymmetry and oculopathy caused by other pathogenesis were also excluded as well as subjects who were unable to cooperate with the inspectors, such as those with diabetic retinal hemorrhage, serious visual impairment, critical disease, and dementia. The study protocol was supported by The 306th Hospital of People's Liberation Army and followed the guidelines of the Declaration of Helsinki. All the participants completed informed consent forms before the study.

### 2.2. Methods

Participant demographic characteristics, history of chronic disease, diabetes duration, clinical characteristics, and current medications were collected via a questionnaire. Anthropometric measurements including height and weight were carried out by a nurse, and BMI was calculated. Patient blood pressure was measured by an electronic sphygmomanometer (OMRON, Japan). Venous blood samples were collected after 8 hours overnight fasting for biochemical examination, including glucose, glycated hemoglobin (HbA1c), total cholesterol, triglyceride (TG), high-density lipoprotein cholesterol (HDL-C), low-density lipoprotein cholesterol (LDL-C), C-reactive protein (CRP), fasting blood glucose (FBG), and fasting insulin (FINS). Postprandial blood glucose and postprandial insulin (PINS) were tested 2 hours after eating snacks.

#### 2.2.1. Eye Examination

All study participants received systematic eye examinations in the ophthalmology clinic of The 306th Hospital of People's Liberation Army. Examination included visual acuity and lens, vitreous body, and fundus exam.

- Visual acuity examination: international standard visual acuity chart with light was applied at a distance of 5 meters. Both eyes were checked. We registered uncorrected visual acuity (UCVA) if the subjects did not wear glasses; if they wore glasses regularly, we tested corrected visual acuity (CVA). According to the World Health Organization criteria, blindness is defined as CVA in any one eye is no more than 0.05 [18]. Results were recorded as 0 = nonblind, 1 = blindness.
- The scanning laser ophthalmoscope (England, panoramic 200) was administered by a trained clinical practitioner to check the condition of the fundus. Image analysis was performed by a professional ophthalmologist. The results were recorded as 0 or 1 (0 = normal, 1 = abnormal). If it fails to distinguish the site of turbidity or bleeding in the refractive media, slit lamp and direct ophthalmoscope were applied.
- Slit lamp exam was performed to check for abnormality of the iris and lens. The iris results were recorded as 0 = normal, 1 = posterior synechia, and 2 = neovascularization. The lens results were registered as 0 = normal, 1 = abnormal.
- Direct ophthalmoscope was used to assess the abnormality of the vitreous. Results were recorded as 0 = normal, 1 = opacity or synchysis, 2 = bleeding, and 3 = organization.

#### 2.2.2. Measurement of Sudomotor Function

Sudomotor nerves, the smallest autonomic sympathetic nerves in the human peripheral nervous system, are long, thin, and unmyelinated C-fibers. These have been shown to be susceptible to damage early in the course of diabetes [13, 19]. SUDOSCAN measures skin conductance based on an electrochemical principle; specifically, the ability of sweat glands to release chloride ions activated by an electrical stimulus, which is a surrogate measure of sudomotor function.

Subjects place their feet and hands on electrode plates (nickel-plated stainless steel sensors). The device applies low, incrementally increasing DC current (<4 V) to the skin of the soles and palms. The current on the electrodes attracts chloride ions in the sweat glands by reverse iontophoresis. At such low voltages, the stratum corneum acts as an insulator, permitting chloride ions to pass solely through the sweat duct [20]; this ensures that only sweat gland function is measured. A time/ampere curve is drawn to calculate ESC (*μ*S). When the C-fibers are damaged, ESC values decrease with the reduction of sweat gland function. An ESC ≥ 60 *μ*S denotes no sweat function impairment, ESC 40–60 *μ*S represents moderate damage, and ESC ≤ 40 *μ*S represents severe damage. In addition, a cardiovascular autonomic neuropathy risk score (CAN-RS) was calculated automatically by integrating age, gender, HbA1c value, and ESC to assess the risk of cardiovascular autonomic dysfunction. CAN-RS ≥ 25% represents risk of CAN.

#### 2.2.3. Statistical Analysis

Statistical analysis was performed by SPSS version 18.0. Continuous variables were presented as mean ± standard deviation, and categorical variables were presented as *n* (%). Student's *t*-test was used to compare the mean difference between binary variables. Conditional logistic regression analysis was conducted to investigate the correlative factors in diabetic oculopathy, and ROC was drawn to evaluate the accuracy of CAN-RS in screening diabetic oculopathy. A two-tailed *P* value of <0.05 was considered significant.

## 3. Results

### 3.1. Demographic Information

Subjects' demographic and clinical characteristics are presented in Table 1. Among the 335 patients, males constituted 59.1%, ages ranged from 18 to 83 years old, and the duration of diabetes was 83.0 ± 73.8 months. The proportion of lens abnormality reached up to 7.8%, and its major clinical manifestation was transparency reduction. The vitreous abnormalities were opacity and synchysis; these accounted for 5.1%. The major abnormal manifestation in the fundus was background diabetic retinopathy (9.9%) which presented as small bleeding dot, hemorrhage blot, hard exudates, and cotton-wool patches.

### 3.2. The Relationship between Autonomic Nerve Function and Ocular Abnormality

Table 2 presents comparisons of the means of CAN-RS, HESC, and FESC between the different ocular abnormalities. The value of CAN-RS was much higher in the lens abnormality group, as well as the vitreous abnormality group, than that in normal ocular patients with T2DM. The differences were statistically significant. For patients with fundus abnormality, the value of CAN-RS was higher than that in normal subjects, but there was no statistically significant difference. The value of HESC and FESC had no statistical difference among the lens, vitreous, and fundus.

### 3.3. Association between CAN-RS Value and Ocular Abnormality

To further investigate the relationship between CAN-RS and ocular abnormality, we conducted conditional logistic regression analysis. The following factors were incorporated into the logistic regression model step by step: CAN-RS, BMI, CRP, total cholesterol, TG, LDL-C, HDL-C, FBG, FINS, postprandial blood glucose, and PINS. The results are listed in Table 3. CAN-RS was positively associated with lens abnormality (OR = 1.055, *P* < 0.001) and vitreous abnormality (OR = 1.044, *P* < 0.01), but not related significantly with fundus abnormality.

LDL-C and PINS could be significant factors that influence fundus abnormality. The risk of abnormal fundus can increase 1.5 times when the LDL-C rose 1 mmol/L, while an increase of 1 unit of PINS led to 1.3% lower risk of fundus abnormality.

### 3.4. The Feasibility and Accuracy of CAN-RS in Screening Ocular Abnormality

**Figure 1:**
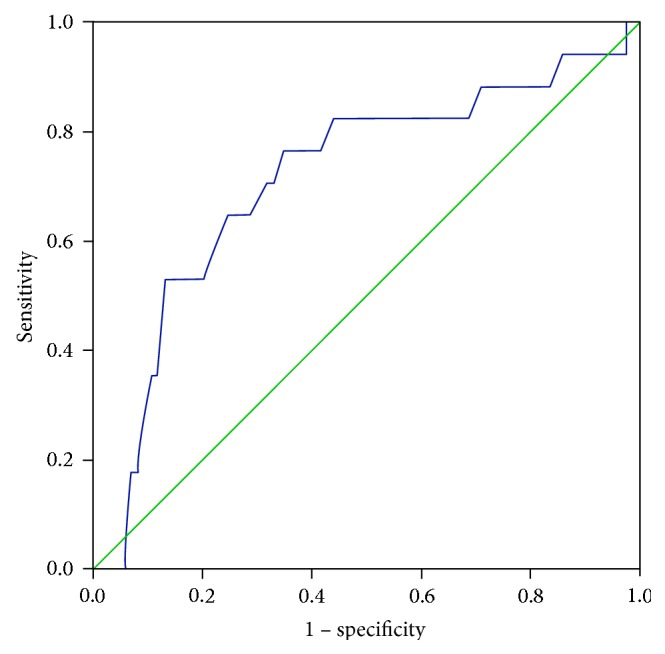
ROC curve for lens abnormality.

**Figure 2:**
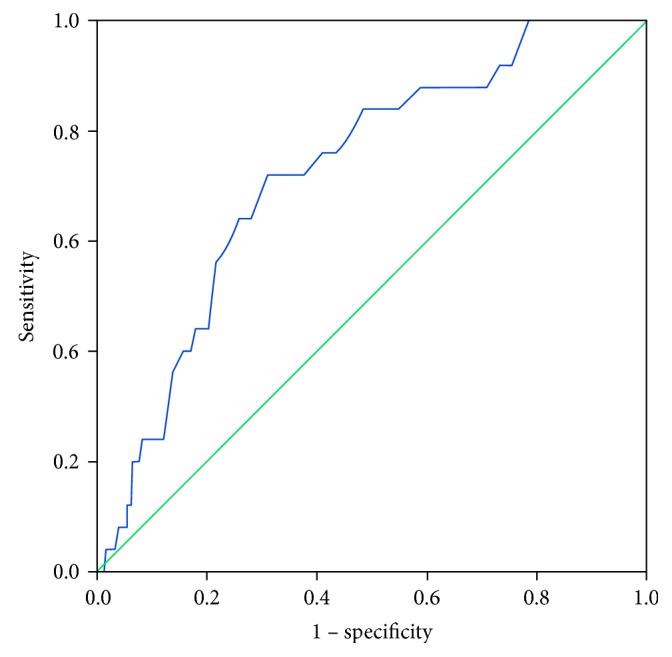
ROC curve for vitreous abnormality.

Participants were categorized using eye examination as the gold standard. Receiver operating characteristic (ROC) curves of CAN-RS were drawn to investigate its feasibility and accuracy in screening for abnormalities of the lens, vitreous, and fundus (seen in Table 4). The optimum cut-off point of CAN-RS for lens abnormality was 37.5%, with a sensitivity of 77% and a specificity of 66%. The area under curve (AUC) was 0.713 (seen in Figure 1). The AUC for detecting vitreous abnormality was 0.725 with a sensitivity of 72% and a specificity of 69% (seen in Figure 2), while the screening efficiency for fundus abnormality was somewhat low with an AUC of 0.537.

## 4. Discussion

The American Association of Clinical Endocrinologists (AACE) recommended sudomotor function as a marker to diagnose early peripheral autonomic neuropathy [19]. SUDOSCAN is a simple, noninvasive method [21–23], and various studies have validated its efficacy in both detecting diabetic peripheral neuropathy (DPN) and CAN screening [12, 15, 21]. In this study, we have demonstrated that ESC of the hands and feet were correlated with CAN-RS (HESC, *r* = −0.342; FESC, *r* = −0.496, *P* < 0.001), and also correlated with resting to standing blood pressure difference (HESC, *r* = −0.192, *P* < 0.001; FESC, *r* = −0.135, *P* = 0.014) in CARTs (these results are not shown in Section 3). Based on this correlation, CAN-RS was applied to investigate the relationship between CAN and diabetic oculopathy.

As for CAN-RS, some investigations have released it as an effective assessment of CAN [14, 15, 24]. A large cross-sectional study involving 4109 people, suggesting that CAN-RS was associated with arterial stiffness independent of traditional risk factors and glucose tolerance [17]. Zhu et al. showed that there was a link between CAN-RS and the incidence and composition of metabolic syndrome [16]. Beyond this, there have been other studies which have indicated that CAN-RS is an effective indicator of CAN and could potentially take the place of the traditional method (CARTs) [13, 14].

For diabetic oculopathy, researchers still focus mainly on diabetic retinopathy (DR). This study expanded its scope into lens and vitreous lesions in addition to abnormalities of the fundus. Our findings suggest that CAN-RS is associated with lens and vitreous abnormality, which may be explained by their pathogenic mechanisms like endothelial dysfunction [25] and polyol pathway [26]. Eranki et al. [27] measured the sensitivity and specificity of CAN-RS for screening at least one microangiopathy (DPN, diabetic nephropathy, and DR) in diabetics; CAN-RS had a sensitivity of 82% and a specificity of 61%, but for retinopathy specifically, the sensitivity was 74% and the specificity was 63%, similar to our report. However, there is no significant correlation between CAN-RS and fundus abnormality in this study (not correlated with orthostatic blood pressure difference too), which was inconsistent with the previous studies. The reason for this discrepancy may lie in the indirect link between CAN and fundus lesions, the difference in enrolled patient population, or the different experimental methods. Significant symptoms of CAN or DPN did not occur in our patients, and fundus lesions were also mild in this study. Further investigation is required to confirm whether CAN-RS is associated with severe fundus lesions like proliferative retinopathy. In addition, a large cross-sectional study recruiting 1736 type 2 diabetic subjects suggested that insulin therapy was an independent risk factor for severity of DR [28]. Since PINS was a protective factor for the lens and fundus in our study, one might suppose that intensive insulin therapy might improve ocular lesions and postpone the onset of cataracts and retinopathy.

The novelty of this research lies in that it took CAN-RS as a new indicator of CAN, instead of traditional methods, and investigated the relationship between CAN and diabetic ocular abnormalities. In addition, this study expanded the scope of diabetic oculopathy research, not limited to fundus lesions. The main finding revealed that CAN-RS correlated with lens and vitreous abnormality, but not with fundus lesions. The data also demonstrated CAN-RS estimated by SUDOSCAN may be a promising index for lens and vitreous abnormality screening in T2DM. However, a prospective study is needed to further confirm whether CAN-RS can be effectively used to screen diabetic ocular abnormality in clinical practice—our research was only an academic exploration.

Our study had some limitations. First, although the SUDOSCAN test has been proven to be a reliable method to assess cardiac autonomic function, and orthostatic blood pressure difference was performed, complete CARTs should be performed. Second, the study sample size was relatively small, and the subjects who completed a variety of eye examinations had few and mild compliances. Therefore, there are some problems and biases in the representation of the general population. Third, for safety, patients were not allowed to stop insulin treatment, hypoglycemic agents, or antihypertensive drugs. We did not assess the influence of drug treatment on the results. Finally, this was a cross-sectional study, and future longitudinal research is needed to validate its conclusions.

In conclusion, our findings suggest that cardiac autonomic nerve dysfunction, measured by CAN-RS, may be correlated with lens and vitreous abnormality in patients with T2DM. Though longitudinal studies are required, CAN-RS could play a promising role in lens and vitreous abnormality screening.

## Figures and Tables

**Table 1:** Demographic and clinical characteristics of the patients with T2DM.

	Whole population
*N*	335
Male, *n* (%)	198 (59.1)
Age (years)	54.7 ± 11.8
Duration of diabetes (months)	83.0 ± 73.8
BMI (kg/m^2^)	26.0 ± 3.5
SBP (mmHg)	127.3 ± 13.5
DBP (mmHg)	77.2 ± 8.1
BPwl (mmHg)	9.56 ± 4.5
HbA1c (%)	8.1 ± 1.9
TG (mmol/L)	1.8 ± 1.5
Total cholesterol (mmol/L)	4.8 ± 1.4
HDL-C (mmol/L)	1.5 ± 0.4
LDL-C (mmol/L)	2.6 ± 0.9
CRP (mg/L)	1.1 ± 2.6
FBG (mmol/L)	14.7 ± 20.4
PBG (mmol/L)	13.3 ± 9.0
FINS (uIU/mL)	14.7 ± 20.4
PINS (uIU/mL)	51.7 ± 49.4
HESC (*μ*S)	74.1 ± 10.4
FESC (*μ*S)	75.7 ± 13.0
CAN-RS	33.1 ± 14.8

Data are mean ± standard deviation for continuous variables and *n* (%) for categorical variables. BMI: body mass index; SBP: systolic blood pressure; DBP: diastolic blood pressure; BPwl: lying to standing blood pressure difference; HbA1c: glycated hemoglobin; TG: triglyceride; HDL-C: high-density lipoprotein cholesterol; LDL-C: low-density lipoprotein cholesterol; CRP: C-reactive protein; FBG: fasting blood glucose; FINS: fasting insulin; PBG: postprandial blood glucose; PINS: postprandial insulin; HESC: hands electrochemical skin conductance; FESC: feet electrochemical skin conductance; CAN-RS: cardiovascular autonomic neuropathy risk score.

**Table 2:** Distribution of autonomic nervous function in normal and abnormal lens, vitreous, and fundus.

	Lens		*P*	Vitreous		*P*	Fundus		*P*
	0	1		0	1		0	1	
CAN-RS	32.3 ± 14.8	43.3 ± 11.6	<0.01^∗^	32.6 ± 14.7	42.0 ± 14.8	0.01^∗^	32.7 ± 14.6	36.5 ± 16.9	0.17
HESC	73.9 ± 10.5	77.3 ± 7.9	0.11	74.1 ± 10.4	74.1 ± 10.1	0.99	73.9 ± 10.5	76.2 ± 9.3	0.21
FESC	76.2 ± 13.0	71.1 ± 13.3	0.06	75.8 ± 13.1	74.3 ± 12.4	0.64	75.6 ± 13.4	77.5 ± 9.2	0.44

Data are mean ± standard deviation. *P* values are Student's *t*-test across the three groups. 0 = normal, 1 = abnormal. CAN-RS: cardiovascular autonomic neuropathy risk score; HESC: hands electrochemical skin conductance; FESC: feet electrochemical skin conductance. ^∗^*P* < 0.05.

**Table 3:** Logistic regression analysis of risk factors associated with different ocular abnormalities.

Dependent variable	Equation variables	*β*	Wald	*P*	OR	95% CI	
						Upper limit	Lower limit
Lens abnormality	CAN-RS	0.054	12.678	<0.001	1.055	1.025	1.087
	PINS	−0.015	4.064	0.044	0.985	0.972	1.000
Vitreous abnormality	CAN-RS	0.043	6.301	0.012	1.044	1.010	1.080
Fundus abnormality	LDL-C	0.434	3.852	0.050	1.543	1.001	2.379
	PINS	−0.013	4.389	0.036	0.987	0.976	0.999

Data are correlation coefficient (*β*), Wald value, *P* for trend, odds ratios, and 95% confidence interval. CAN-RS: cardiovascular autonomic neuropathy risk score; LDL-C: low-density lipoprotein cholesterol; PINS: postprandial insulin.

**Table 4:** ROC curve of the CAN-RS for abnormality in the lens, vitreous, and fundus.

	AUC	Sensitivity	Specificity	Cut-off value	95% CI of ACU	
					Lower	Upper
Lens abnormality	0.713	0.77	0.66	37.5	0.632	0.819
Vitreous abnormality	0.725	0.72	0.69	39.5	0.577	0.850
Fundus abnormality	0.537	0.34	0.82	46.5	0.457	0.689
